# Supplementary material for: Salinity causes widespread restriction of methane emissions from small inland waters
Source: Nat Commun. 2024 Jan 24;15:717. doi: 10.1038/s41467-024-44715-3 (PMC10808391; doi:10.1038/s41467-024-44715-3)
Supplement: Supplementary file 1 — Supplementary Information [file 41467_2024_44715_MOESM1_ESM.pdf]

Supporting Information For:

Salinity causes widespread restriction of methane emissions from small inland waters

Authors:

Cynthia Soued[1 †], Matthew J. Bogard[1†*], Kerri Finlay[2, 3], Lauren E. Bortolotti[4], Peter R. Leavitt[3,5], Pascal Badiou[4], Sara H. Knox[6], Sydney Jensen[2], Peka Mueller[1], Sung Ching Lee[6], Darian Ng[6], Björn Wissel[3,7], Chun Ngai Chan[1], Bryan Page[4], Paige Kowal[4]

†Authors contributed equally to this manuscript.

Content:

- **Table S1:** Summary statistics, mean, standard deviation (SD), range (min and max), number of observations (n) of measured variables by system type.

- **Table S2.** Estimates of variance (proportion of total variance given in brackets) associated with the random effects from a lognormal generalized linear mixed model of pCH_4 in wetland ponds for each Prairie province (AB = Alberta, SK = Saskatchewan, MB = Manitoba). The wetlands were sampled in the 2021 peripheral survey (Fig. S1). The approach, justification, and interpretation of results is fully detailed in the supporting text (Supporting Text S2).

- **Table S3.** Mean estimated marginal means (emmeans) and standard errors from simulations of differential allocation of sampling effort using results from a lognormal generalized linear mixed model of pCH_4 as a function of province (AB = Alberta, SK = Saskatchewan, MB = Manitoba), with random effects of site(province), station(province*site), and date(site*station). Maximum number of sampling dates differ by province to match actual sampling effort in the source data and all simulations were constrained to maximum 471 total samples to match source data. The approach, justification, and interpretation of results is fully detailed in the supporting text (Supporting Text S2).

- **Table S4.** Environmental conditions at the high- and low-salinity wetland eddy-covariance measurement sites. Where multiple measurements were taken from May to October, 2021, an average and standard deviation value is provided. For each site, a total of 40 observations were made for water quality parameters in 2021.

- **Fig. S1.** Map of study sites in the Canadian Prairie Provinces. The map includes primary sampling sites (where all parameters were measured), peripheral wetland pond sampling sites (where pCH_4 and some water quality parameters were measured to constrain uncertainty in our scaling effort; see inset boxes), and wetland eddy covariance (EC) flux tower sites each at a high- and low salinity wetland (stars in inset boxes). For the primary sampling sites, density distribution plots of measured environmental properties are presented by ecosystem type. See Table S1 for summary statistics.

- **Fig S2.** Results of multiple linear regression predictions of pCH_4 as a function of limnological variables. Analyses were grouped either by lakes, rivers, or small (<0.1 km^2) lentic systems represented by wetlands and ponds. Analyses are performed on log_{10} transformed data and standardized to a standard deviation of 1 to compare regression coefficients (effect size). Circle size represents the effect size of each explanatory variable, and the green and red colors represent positive vs negative effects, respectively.

The light to dark color gradient is scaled to a 0 to 1 confidence value around the effect size, computed as 1 – SE/Eff, with Eff the effect size and SE its standard error, with negative values (SE > Eff) considered as 0. Regression statistics are reported in Table 1.

- **Fig. S3.** Interaction between nutrients, organic matter, and salinity as predictors of pCH_4. Comparison of the relationship between $\log_{10}$ values of pCH_4 and either the ratio of DOC concentration (mg L^{-1}) to salinity, or total phosphorus (TP; μg L^{-1}) concentration to salinity, or total nitrogen concentration (TN; mg L^{-1}) to salinity for primary study sites including all small lentic systems (wetlands and small agricultural ponds). Pearson correlation (*r*) and associated probability statistics are listed for each relationship.

- **Fig. S4.** Peripheral survey sites show consistent trends with primary sites. The relationship between $\log_{10}$ transformed values of both pCH_4 and the ratio of dissolved organic carbon concentration (mg L^{-1}) to salinity (ppt) at peripheral pond wetland sampling sites. Linear regression model: p = << 0.001; R^2 = 0.26; slope = 1.264; intercept = 0.0028.

- **Fig S5.** Salinity versus sulfate concentration in the three types of sampled lentic systems that were part of the primary sampling sites. The linear regression yields a p-value << 0.001 and a R^2_{adj} = 0.56.

- **Fig. S6.** Exploring salinity versus SO_4^{2-} concentration as predictors of pCH_4. Comparison of the relationship between $\log_{10}$ pCH_4 and either the ratio of DOC concentration (mg L^{-1}) to salinity, or DOC concentration to SO_4^{2-} concentration (mg L^{-1}) for primary study sites including all lentic systems combined, and individual relationships for lakes, wetlands and small agricultural ponds. Pearson correlation (*r*) and associated probability statistics are listed for each relationship.

- **Fig. S7.** Continuous eddy-covariance measurements of CH_4 emissions during the ice-free period from two wetland ecosystems. Each site in in Manitoba, Canada, is broadly representative of hardwater (salt rich) and soft water habitats in the Canadian Prairie Pothole region.

- **Fig S8.** Increasing SO_4^{2-} concentrations in recent decades in most monitoring lake sites. Map depicting long-term (1990-2020) trends (Sen slopes) in inland water SO_4^{2-} concentration in monitored sites in the Canadian Prairies, based on publicly available data from the Saskatchewan Water Security Agency.

- **Fig. S9.** Salinity scales inversely with average ebullition flux rates (error bars ± 1 S.D.). Open water measurements from agricultural ponds shown in figure 2. Note $\log_{10}$ scale of both axes.

- **Fig. S10.** The relationship between log_{10} transformed SO_4^{2-} and Cl^- concentrations in diverse global saline systems. Data replotted from Table 3 of Deocampo and Jones[1].

- **Supporting Text S1.** Expanded discussion on the use of salinity versus SO_4^{2-} content as predictors of CH_4 cycling.

- **Supporting Text S2.** Analysis exploring the importance of within-site, cross-season, and cross-site replication for improving estimates of pCH_4.

Supporting Tables:

Table S1: Summary statistics, mean, standard deviation (SD), range (min and max), number of observations (n) of measured variables by system type.

Variable	System type	Mean	Median	SD	Min	Max	n
Area (km^2)	All	7.085	0.0015	47.4831	0.0001	500	169
	Rivers						0
	Lakes	70.099	8.6	137.7732	0.5	500	17
	Wetlands	0.1229	0.0056	0.7454	0.0001	5.01	45
	Ponds	0.0014	0.001	0.0018	0.0002	0.0139	107
Max depth (m)	All	2.83	2.1	3.41	0.12	27.7	148
	Rivers						0
	Lakes	9.46	9	6.4	2.8	27.7	17
	Wetlands	1.08	0.7	2.01	0.12	10	23
	Ponds	2.15	2.1	0.97	0.18	5.1	108
Temperature (°C)	All	20.1	20.2	2.6	13	29.5	193
	Rivers	19	18.5	3	13	23.9	23
	Lakes	20.3	20.5	1.4	17.6	22.3	17
	Wetlands	20.7	20.4	3	15.6	26.8	45
	Ponds	20.1	20	2.5	15.7	29.5	108
pH	All	8.6	8.62	0.71	6.76	10.5	193
	Rivers	8.33	8.35	0.36	7.3	9.34	23
	Lakes	8.82	8.8	0.21	8.49	9.14	17
	Wetlands	8.29	8.29	0.94	6.76	9.9	45
	Ponds	8.75	8.72	0.65	6.95	10.5	108
Salinity (ppt)	All	0.95	0.45	1.55	0.07	13.27	193
	Rivers	0.28	0.16	0.26	0.1	0.92	23
	Lakes	1.48	0.79	1.86	0.12	5.93	17
	Wetlands	1.33	0.53	2.39	0.08	13.27	45
	Ponds	0.84	0.45	1.12	0.07	8.57	108
Sp. Conductivity ($\mu S\ cm^{-1}$)	All	1656	838	2446	136	18974	193
	Rivers	520	345	421	201	1549	23
	Lakes	2749	1579	3263	263	10494	17
	Wetlands	2226	995	3555	162	18974	45
	Ponds	1489	810	1811	136	13489	108
SO_4 ($mg\ L^{-1}$)	All	1037.9	558.45	1454.74	0.72	9489.78	118
	Rivers						0
	Lakes	1273.54	533.3	1823.34	78.55	6304.24	14
	Wetlands	804.92	293.76	1274.02	0.72	6686.67	42
	Ponds	1142.51	703.81	1482.69	0.99	9489.78	62
DOC ($mg\ L^{-1}$)	All	28.2	25.5	20.1	0.4	126.9	190
	Rivers	5.8	2.3	7.5	0.4	25.6	23
	Lakes	18	17.3	10.4	4.4	43.6	17
	Wetlands	36.5	31.2	26.2	3.1	126.9	45

	Ponds	31.3	29.1	15.7	4.6	90.4	105
TP (µg L^{-1})	All	267	80	621	1	6480	189
	Rivers	154	20	519	1	2508	23
	Lakes	143	71	171	12	539	17
	Wetlands	330	117	543	30	2540	45
	Ponds	285	80	713	9	6480	104
TN (mg L^{-1})	All	2.48	1.95	2.07	0.05	14.28	189
	Rivers	0.54	0.27	0.66	0.05	3.05	23
	Lakes	1.44	1.43	0.68	0.48	3.25	17
	Wetlands	2.65	1.96	1.69	0.99	9.54	45
	Ponds	3	2.3	2.26	0.42	14.28	104
DOC / salinity (mg L^{-1} ppt^{-1})	All	59.7	43.8	60.8	2.4	410.3	190
	Rivers	17.7	14.4	16.9	2.4	78.3	23
	Lakes	21.9	19.5	12.4	5.4	46.9	17
	Wetlands	75	53	88	5.2	410.3	45
	Ponds	68.5	56.5	50.6	8	276.9	105
pCH_4 (ppm)	All	2653	933	4904	2	40882	191
	Rivers	481	160	685	5	2750	23
	Lakes	412	163	778	2	3225	16
	Wetlands	4038	1742	6028	52	30244	45
	Ponds	2873	1324	5020	34	40882	107
CH_4 diffusion (mmol m^{-2} d^{-1})	All	6.12	2.76	10.6	0.11	91.47	139
	Rivers						0
	Lakes						0
	Wetlands	3.42	1.75	4.13	0.13	14.11	20
	Ponds	6.58	2.9	11.28	0.11	91.47	119
CH_4 ebullition (mmol m^{-2} d^{-1})	All	0.89	0.03	1.65	0	4.66	10
	Rivers						0
	Lakes						0
	Wetlands	1.12	0.01	2.02	2×10^{-6}	4.66	5
	Ponds	0.67	0.05	1.38	3×10^{-4}	3.13	5

Table S2. Estimates of variance (proportion of total variance given in brackets) associated with the random effects from a lognormal generalized linear mixed model of pCH_4 in wetland ponds for each Prairie province (AB = Alberta, SK = Saskatchewan, MB = Manitoba). The wetlands were sampled in the 2021 peripheral survey (Fig. S1). The approach, justification, and interpretation of results is fully detailed in the supporting text (Supporting Text S2).

Variance Component	AB Estimate (Proportion of Variance)	SK Estimate (Proportion of Variance)	MB Estimate (Proportion of Variance)
Site	0.66 (0.37)	3.85 (0.77)	1.78 (0.61)
Station(Site)	0.00 (0.00)	0.00 (0.00)	0.00 (0.00)
Date(Site*Station)	1.12 (0.63)	1.12 (0.23)	1.12 (0.39)

Table S3. Mean estimated marginal means (emmeans) and standard errors from simulations of differential allocation of sampling effort using results from a lognormal generalized linear mixed model of pCH_4 as a function of province (AB = Alberta, SK = Saskatchewan, MB = Manitoba), with random effects of site(province), station(province*site), and date(site*station). Maximum number of sampling dates differ by province to match actual sampling effort in the source data and all simulations were constrained to maximum 471 total samples to match source data. The approach, justification, and interpretation of results is fully detailed in the supporting text (Supporting Text S2).

Province	Mean Emmeans	Mean Std Err	No. of Sites	Max. Dates (AB)	Max. Dates (SK)	Max. Dates (MB)
AB	5.836	0.218	16	9	7	14
SK	4.214	0.476	16	9	7	14
MB	4.851	0.309	16	9	7	14
AB	5.857	0.173	32	5	4	7
SK	4.331	0.359	32	5	4	7
MB	4.885	0.240	32	5	4	7

Table S4. Environmental conditions at the high- and low-salinity wetland eddy covariance measurement sites. Where multiple measurements were taken from May to October, 2021, an average and standard deviation value is provided. For each site, a total of 40 observations were made for water quality parameters in 2021.

	Site Name	Province	Site I.D. code	Surface Area (Ha)	Salinity (psu)	pH	Specific Cond. (uS/cm)	DOC (mg/L)	TDN (mg/L)	TP (ug/L)	SO_4^{2-} (mg/L)
Mean	Young	MB	MB15	14.5	0.57	8.95	982	28.93	2.32	494.3	320.0
S.D.	Young	MB	MB15		0.14	0.52	209	4.96	0.89	252.5	90.4
Mean	Hogg	MB	MB16	20.3	2.31	8.70	3576	86.86	4.87	164.4	1114.2
S.D.	Hogg	MB	MB16		0.76	0.25	1043	26.46	1.64	74.1	343.8

Supporting Figures:

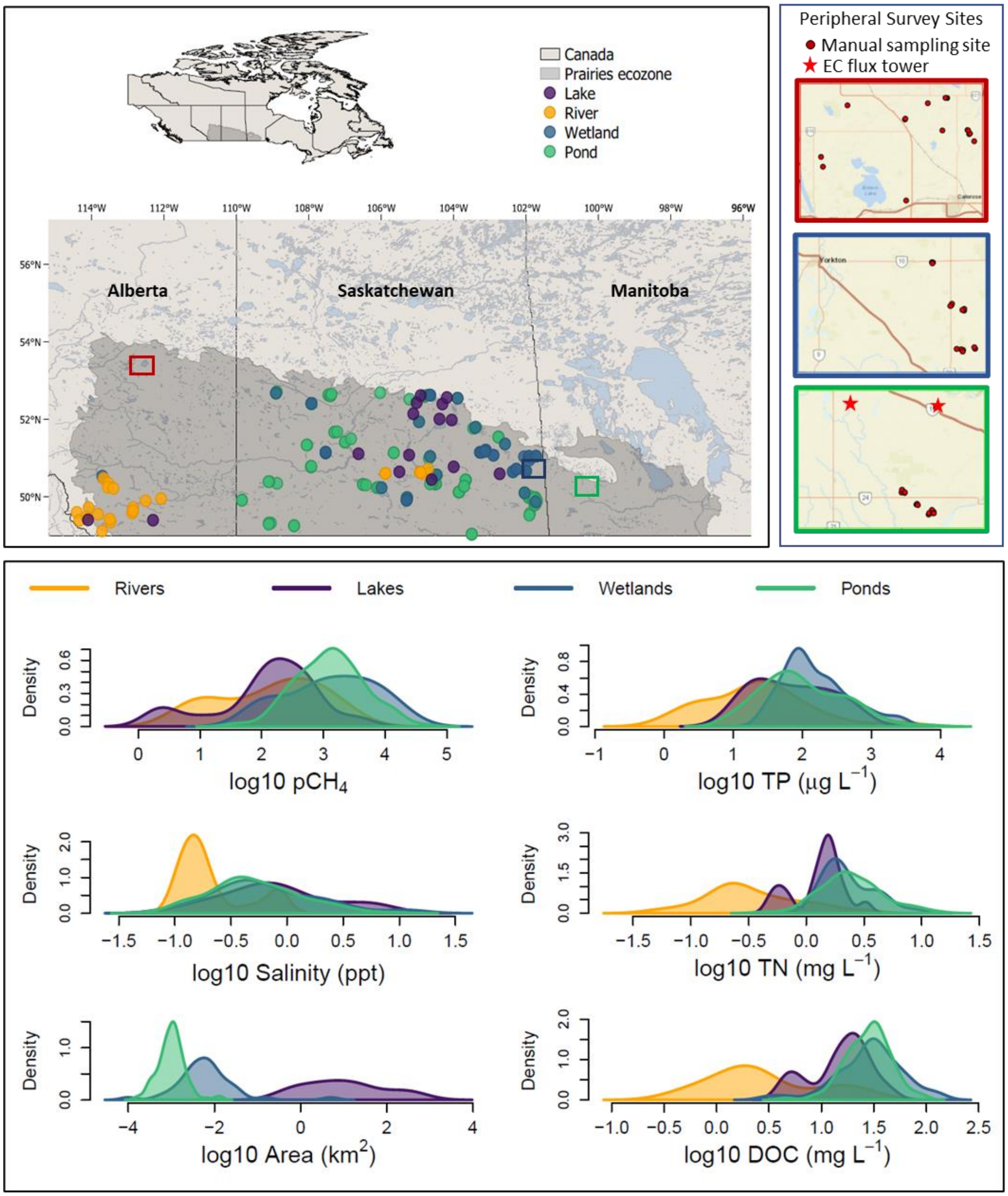

Fig. S1. Map of study sites in the Canadian Prairie Provinces. The map includes primary sampling sites (where all parameters were measured), peripheral wetland pond sampling sites (where pCH_4 and some water quality parameters were measured to constrain uncertainty in our scaling effort; see inset boxes), and wetland eddy covariance (EC) flux tower sites each at a high- and low salinity wetland (stars in inset boxes). For the primary sampling sites, density distribution plots of measured environmental properties are presented by ecosystem type. See Table S1 for summary statistics.

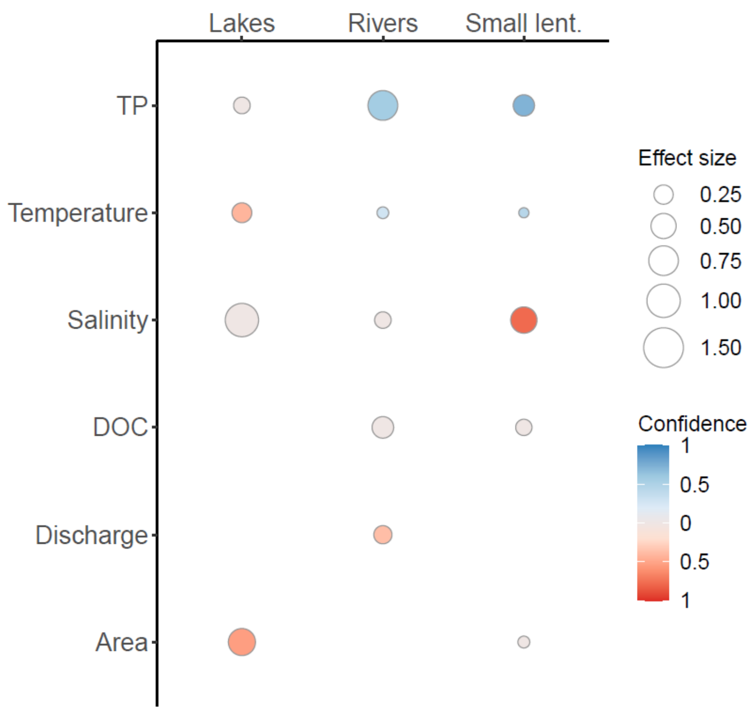

Fig S2. Results of multiple linear regression predictions of pCH_4 as a function of limnological variables. Analyses were grouped either by lakes, rivers, or small (<0.1 km^2) lentic systems represented by wetlands and ponds. Analyses are performed on log_{10} transformed data and standardized to a standard deviation of 1 to compare regression coefficients (effect size). Circle size represents the effect size of each explanatory variable, and the green and red colors represent positive vs negative effects, respectively. The light to dark color gradient is scaled to a 0 to 1 confidence value around the effect size, computed as 1 – SE/Eff, with Eff the effect size and SE its standard error, with negative values (SE > Eff) considered as 0. Regression statistics are reported in Table 1.

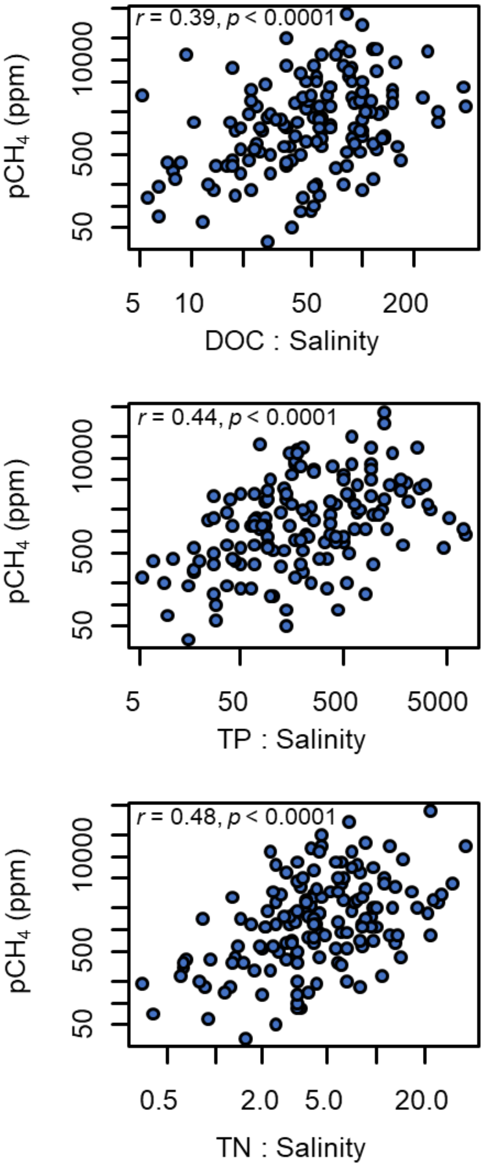

Fig. S3. Interaction between nutrients, organic matter, and salinity as predictors of pCH_4. Comparison of the relationship between log_{10} values of pCH_4 and either the ratio of DOC concentration (mg L^{-1}) to salinity, or total phosphorus (TP; µg L^{-1}) concentration to salinity, or total nitrogen concentration (TN; mg L^{-1}) to salinity for primary study sites including all small lentic systems (wetlands and small agricultural ponds). Pearson correlation (*r*) and associated probability statistics are listed for each relationship.

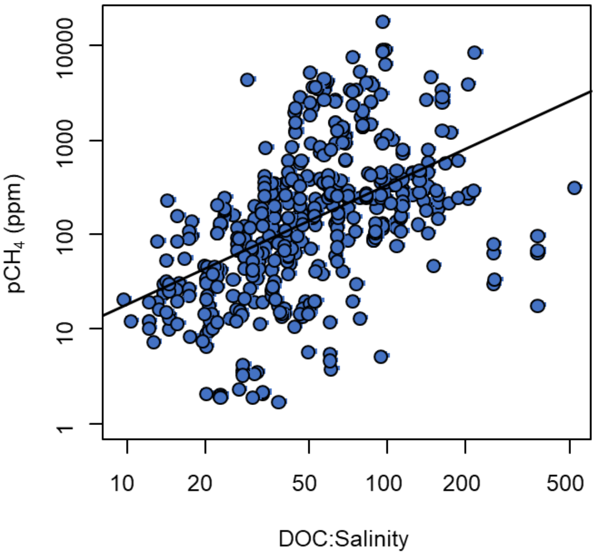

Fig. S4. Peripheral survey sites show consistent trends with primary sites. The relationship between $\log_{10}$ transformed values of both pCH_4 and the ratio of DOC (mg L^{-1}) / salinity (ppt) at peripheral pond wetland sampling sites. Linear regression model: p-value << 0.0001; R^2adj = 0.25; slope = 1.264; intercept = 0.0028, n = 465.

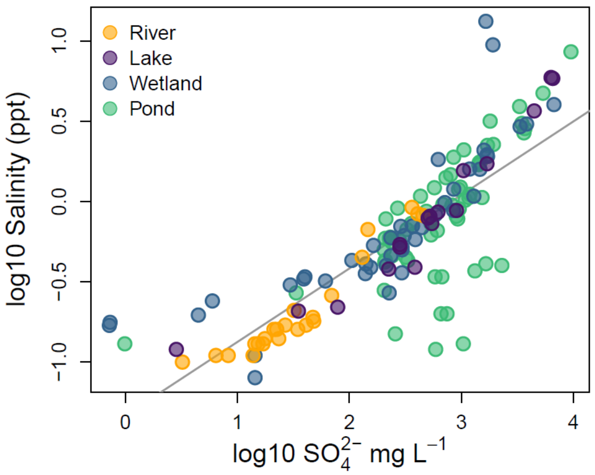

Fig S5. Salinity versus sulfate concentration in the three types of sampled lentic systems and the rivers that were part of the primary sampling sites. The linear regression yields a p-value << 0.001 and a R^2_{adj} = 0. 68.

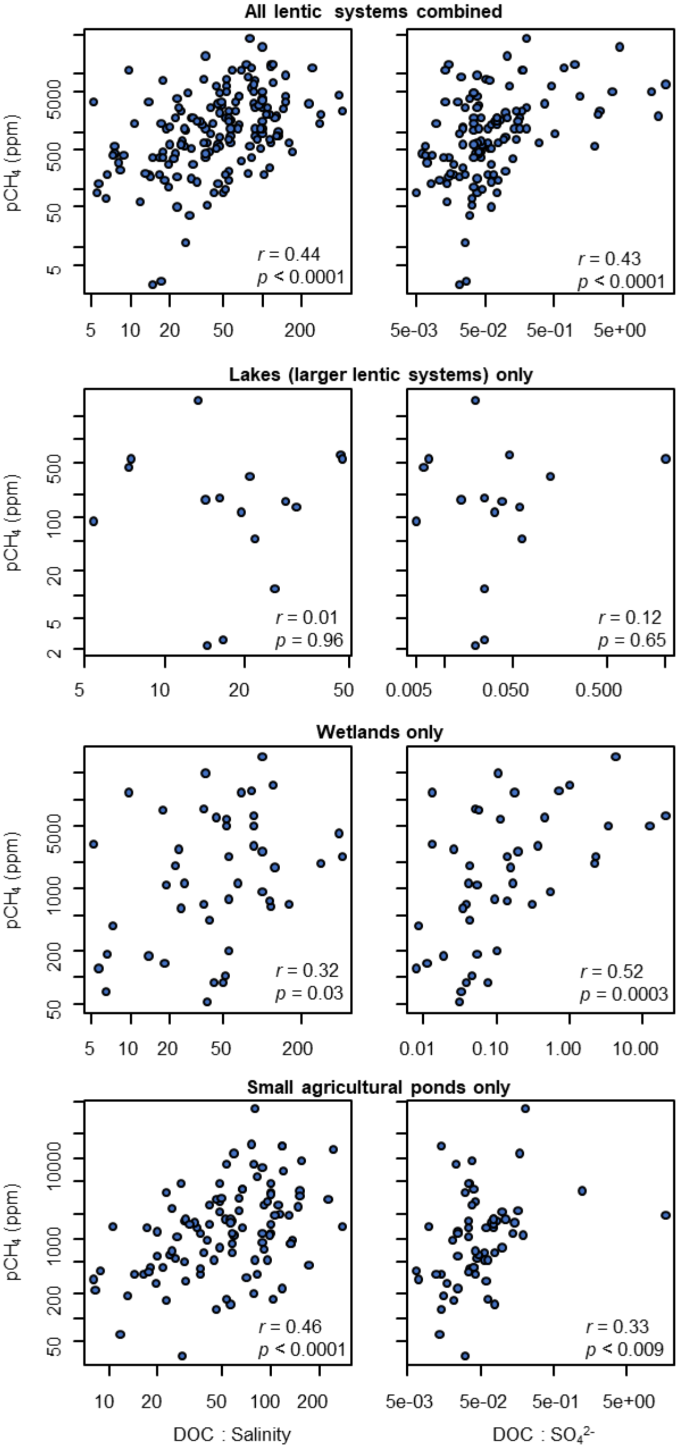

Fig. S6. Exploring salinity versus SO_4^{2-} concentration as predictors of pCH_4. Comparison of the relationship between $\log_{10}$ pCH_4 and either the ratio of DOC concentration (mg L^{-1}) to salinity, or DOC concentration to SO_4^{2-} concentration (mg L^{-1}) for primary study sites including all lentic systems combined, and individual relationships for lakes, wetlands and small agricultural ponds. Pearson correlation (r) and associated probability statistics are listed for each relationship.

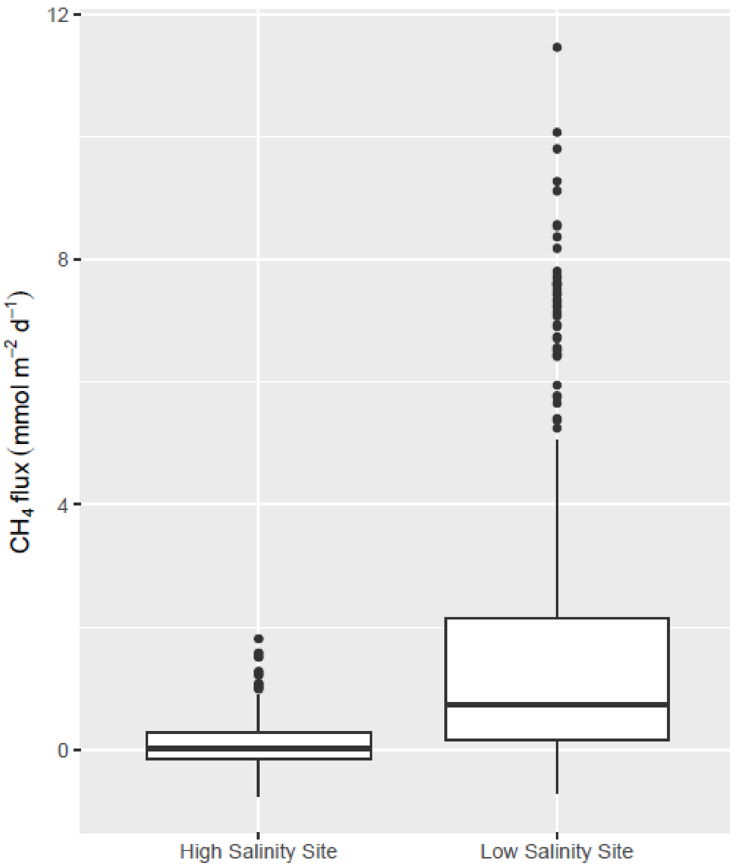

Fig. S7. Continuous eddy-covariance measurements of CH_4 emissions during the ice-free period from two wetland ecosystems. Each site in in Manitoba, Canada, is broadly representative of hardwater (salt rich) and soft water habitats in the Canadian Prairie Pothole region.

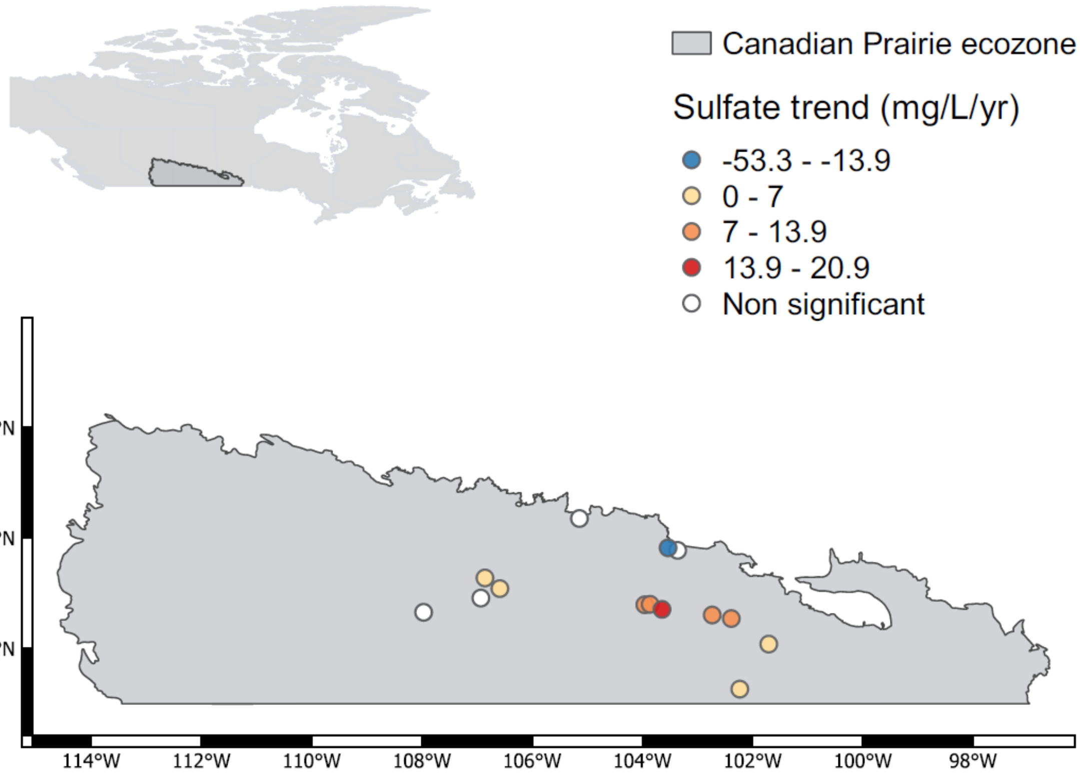

Fig. S8. Increasing SO_4^{2-} concentrations in recent decades in most monitoring lake sites. Map depicting long-term (1990-2020) trends (Sen slopes) in inland water SO_4^{2-}concentration in monitored sites in the Canadian Prairies, based on publicly available data from the Saskatchewan Water Security Agency.

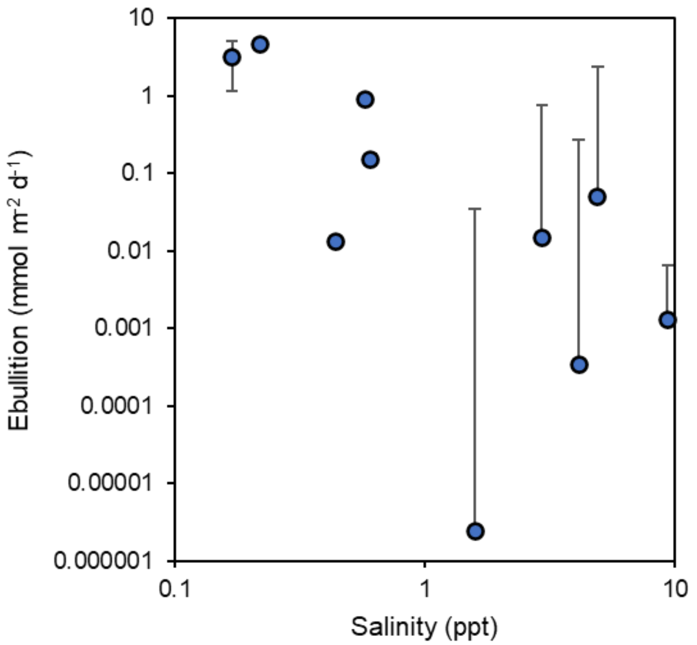

Fig. S9. Salinity scales inversely with average ebullition flux rates (error bars ± 1 S.D.). Open water measurements from agricultural ponds shown in figure 2. Note $\log_{10}$ scale of both axes.

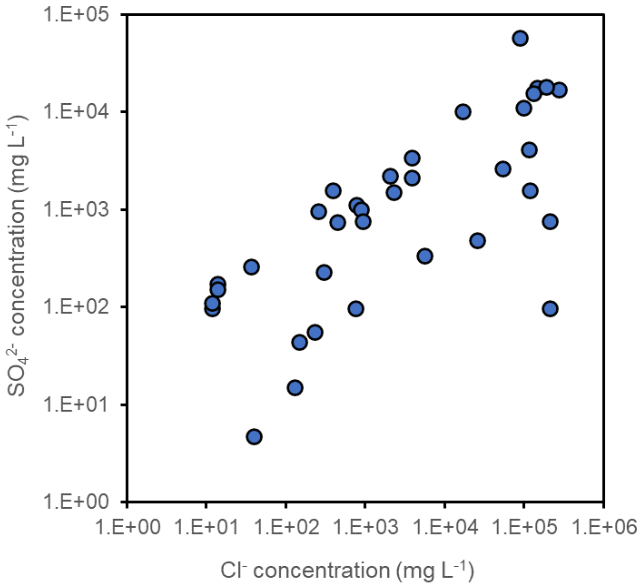

Fig. S10. The relationship between $\log_{10}$ transformed SO_4^{2-} and Cl^- concentrations in diverse global saline systems. Data replotted from Table 3 of Deocampo and Jones[1].

Supporting Text:

Supporting Text S1. Expanded discussion on the use of salinity versus individual ions as predictors of CH_4 cycling.

Below we justify the use of salinity as a predictor of CH_4 content and fluxes in place of other predictors, namely individual ions such as SO_4^{2-} content. Previous research on inland water CH_4 cycling has relied on different measurements including more general indicators of ionic strength (salinity, specific conductance) versus the use of SO_4^{2-} concentrations alone. Sulfate is often the most abundant anion in many landscapes and is the best studied and probably most important ion that sets redox conditions that favour alternate processes over methanogenesis. However, as detailed below, the cycling of S is not the only mechanism controlling CH_4 and restricting CH_4 content and emissions at elevated salinity. Here, our objective was to deliver the broadest, overarching empirical assessment of the factors that control CH_4 cycling in hardwater environments. Thus, we used salinity to establish these empirical relationships because this metric represented an important gap in the inland water literature that would serve to establish broad relationships which future studies can refine. Below, we justify this approach by demonstrating that measures of salinity capture multiple factors that impact the CH_4 cycle, each likely varying in relative importance from one system or region to another.

Using salinity as a broad and integrative predictor:

Distinct ecosystems, or even regions[1,2] may have different geochemistry and surface water ionic composition that in turn drives the gradient in regional salinity (from freshwater to

sub- and saline systems). These differences will in turn make salinity a predictor that, when comparing ecosystems among distinct geochemical regions, can reflect variable ionic compositions of surface waters. Therefore, the use of salinity as a predictor in our study is akin to the widespread prediction of ecosystem structure and functioning based on measurements of other general chemical features, such as the use of total phosphorus (TP)[3–5], total nitrogen (TN)[5–7], dissolved organic carbon (DOC)[8,9] concentrations, or ratios of these predictors[6,10,11] to predict broad patterns in ecosystem features. While it is known that the chemical composition of the pool of nutrients and C making up each bulk chemical measurement will vary from one ecosystem to the next, all of these widely-used predictors are useful because they provide general indications of the scaling of ecosystem features and functioning. Foundational relationships across aquatic ecosystems have been established with these metrics, all the while with the knowledge that site- or regional variations in the shapes of these relationships are present (e.g., [12,13]). This knowledge does not preclude or invalidate the use of bulk chemical metrics but compliments it. Here, the use of salinity represents a comparable step toward generating predictive tools to help explain CH_4 fluxes at the global scale. More narrow explorations of individual ions, and how local or regional differences in CH_4 content and flux may vary with salinity as ionic composition shifts, represents a more focused and complementary avenue of exploration. Broadly, salinity is a useful predictor of overall availability of elements that interact with CH_4 cycling, because major ions generally scale in concentration in a positive way (see Fig. S10 for SO_4^{2-} and Cl^- for a global relationship in saline systems[1]), and trace element content generally scales with other ions[14]. Therefore, our proposed use of salinity represents the upper-most tier of a hierarchical approach to exploring CH_4 relationships with major ions. Such an approach requires that we establish the broad, generalizable relationships to salinity (much like

has been done with TN or TP and metrics of trophic status or food web features), while also exploring relationships with individual ions or elements. This framework will provide the most comprehensive understanding of the CH_4 cycle in hardwater ecosystems.

Salinity is a predictor of CH_4 concentration and flux in non-SO_4^{2-} dominated hardwaters:

We take this line of reasoning (that salinity is a consistently useful metric to predict CH_4 cycling in hardwater landscapes) a step further with a demonstration that salinity can reflect diverse mechanisms of suppressed CH_4 production, even in systems where ionic composition is vastly different from the SO_4^{2-} dominated ecosystems in the Canadian Prairies. While the global relationship between SO_4^{2-} and Cl^- is positive (Fig. S10), local variation in this pattern exists. The ratio of dissolved SO_4^{2-} to Cl^- content is typically elevated in the northern Great Plains region of North America (~ 1 to 13) relative to other regions where the values typically are < 1, reflecting previously detailed differences in the evolution of contemporary brine composition and dissolved major ion abundances[2]. Yet despite these regional differences in brine composition, extremely restricted rates of CH_4 production are seen at elevated salinities even in regions where SO_4^{2-} is a less important contributor to salinity.

We present published observations from hardwater ponds and lakes from the Iberian Peninsula that both demonstrate this point and add yet another region to our study that is consistent with our conclusions derived from figure 2 (despite the lack of appropriate data to add the systems to that figure). The suppression of CH_4 production has been documented in Spanish lakes that have distinct geochemical properties from most of our study systems[15,16]. As demonstrated by Margalef-Marti et al.[15], Gallocanta Lake has SO_4^{2-} to Cl^- ratios ~ < 0.01

(approximated from the range of SO_4^{2-} concentrations reported for surface waters, and reports of Cl^- concentrations from previous work cited therein) and receives SO_4^{2-} -poor inputs from inflowing surface water. Yet, the abundance of other ions that complex with SO_4^{2-} ultimately enhances the availability of SO_4^{2-} to microbes in surface sediments via the dissolution of mineral-bound SO_4^{2-}. This mechanism appears to fuel local S cycling via coupling to the cycling of other reactive elements, and ultimately restricts CH_4 production and emissions[15] (mechanisms detailed below). As a second example from this region, Camacho et al.[16] demonstrate that the enhanced availability of NaCl, or dilution of salt-rich surface water (using pure water) can respectively suppress or enhance CH_4 production in sediment incubations from lakes in the region. This study did not pinpoint the exact mechanisms that led to the observed salinity dependence of CH_4 production, though it demonstrates that the net sediment CH_4 production that underpins both diffusive and ebullitive emissions is heavily dependent on general ecosystem salinity, in a non-linear manner consistent with our empirical observations (Fig. 1c). Possible insight into the mechanisms underlying NaCl impacts in incubations by Camacho et al.[16] may be taken from a similar incubation experiment where NaCl additions to Australian wetland sediment also restricted net CH_4 production through multiple mechanisms[17]. There, NaCl availability liberated reduced compounds (Fe^{2+}, Mn^{2+}, NH_4^+) that could stimulate anaerobic CH_4 oxidation (detailed below), while suppressing methanogenesis[17]. Taken together, these studies demonstrate that even in non-SO_4^{2-} dominated systems in other global regions, increases in salinity can suppress CH_4 emissions even without the direct modification of SO_4^{2-} loading. Further, the examples outlined here clearly demonstrate that salinity as a metric provides critical information about ecosystem CH_4 cycling. Had we assumed that CH_4 production only proceeds in SO_4^{2-} -rich environments and does not extend to hardwater systems with comparatively low

SO_4^{2-} content, we might erroneously assume elevated CH_4 content and emissions from SO_4^{2-} - poor systems that apparently have other factors (discussed below) that may be regulating emissions.

Salinity captures diverse drivers of microbial control (redox, salt stress):

Salinity is useful because it integrates many complex processes at once that may interact to shape CH_4 cycling. Elevated salinity typically reflects an increase in ecosystem pH, which can dramatically shift the redox state of a given habitat toward more oxidizing conditions[18,19]. More saline shallow systems are typically prone to partial or complete desiccation (seasonally or for extended periods) that also expose shallow sediment layers to the atmosphere and shift these habitats to oxidizing conditions that may favour aerobic degradation of organic matter while inhibiting methanogenesis[16]. Limits on methanogenesis in sediments due to redox properties are closely related to SO_4^{2-} concentrations[20], which can be directly or indirectly[15] enhanced at elevated salinity. Competition for binding sites can liberate NH_4^+ or Fe^{2+} when ions including Na^+ are abundant with increasing salinity (ref.[17] and references therein). As salinity increases, the direct impacts of osmotic stress on bulk microbial communities are complex[17], and halotolerant organisms can increasingly dominate and sustain microbial metabolism. Yet some archaeal methanogens are sensitive to salt stress and show decreased metabolic activity with increasing salinity as NaCl, even at low concentrations (500 $\mu S\ cm^{-1}$ specific conductance)[17].

The role of diverse elements in anaerobic oxidation of CH_4 (AOM):

Enhanced AOM may be another mechanism restricting sediment CH_4 release in hardwaters. Recent discoveries suggest that the pathways supporting AOM may be more diverse in inland waters than marine systems[21] (but see ref.[22]). As reviewed elsewhere[23,24] the prevailing assumption has been that SO_4^{2-} was the only oxidant that fuelled AOM in inland waters. Intense AOM has been documented in inland water anoxic habitats[23,24] and importantly restricts surface water CH_4 content and emissions. At present, the biochemical pathways and organisms that drive inland water AOM are not well established, with new organisms and mechanisms discovered in recent years (e.g.,[25–27]), and unidentified controls yet to be established[24]. However, it is now abundantly clear that the availability and involvement of non-S-based forms of oxidized compounds, including trace metals must be considered in many cases to account for elevated AOM rates that cannot be accounted for by the reduction of SO_4^{2-} alone[24]. As reviewed elsewhere[23,24,28], microbial consumption of CH_4 has now been shown to be coupled to the reduction of diverse electron acceptors that to date are known to include Mn(IV) (ref.[21,25]), Fe^{3+} (ref.[21]), NO_2^- and NO_3^{2-} (ref.[21,27]), Cr(VI)[26], and complex dissolved organic matter[29]. New research in marine environments also suggests that considerable AOM may in some cases proceed independently of SO_4^{2-} reduction[22]. These discoveries are important in the context of using salinity to predict CH_4 cycling, because gradients of salinity reflect the abundance of substrates that are now known to act as electron acceptors in diverse AOM pathways, including minerals rich in metals and trace metals[1,14], and the liberation of ionized, reduced N and Fe from sediment that are precursors for substrates used in AOM (e.g., ref[17]) under saline conditions. Furthermore, and of relevance to hardwater ecosystems, elevated surface water N content is both a natural and anthropogenically enhanced feature in these landscapes[16,30,31]. Additional evapo-concentration of this N alongside other important substrates may provide substrate to drive

AOM. Consequently, the availability of N could be heavily implicated in AOM through diverse pathways (detailed above) that decouple the process from S cycling and may partially account for different empirical relationships between metrics of CH_4 cycling and salinity versus SO_4^{2-} alone. When we take all these lines of evidence together, it is clearly possible that inverse correlations between salinity and CH_4 content and emissions rates may partly reflect an increasing importance of a diversity of AOM pathways restricting net CH_4 production that may not always be captured in regressions with SO_4^{2-} content alone. While more focused research is required, this proposed mechanism may partially explain why empirical relationships with salinity were stronger than SO_4^{2-} content as predictors of pCH_4 in the agricultural reservoirs (Fig. S6), and why salinity remains a strong predictor of pCH_4 in other regions with diverse geologies[16,17] (Fig. 2).

Salinity can alter abiotic conditions that modify CH_4 cycling:

Elevated salinity can importantly modify the physico-chemical conditions in surface water (e.g., alkalization) and sediment layers (e.g., deposition of complex mineral precipitates) in complex ways that lead to suppressed CH_4 release. As salinity increases, individual ions (not necessarily SO_4^{2-}) with greater binding capacities can replace N bound in sediment complexes, and liberate NH_4^+ which is a precursor to important terminal electron acceptors (NO_3^{2-} and NO_2^-) that fuel AOM (detailed above). In hardwater environments, complexation and precipitation of substrates (metals, nutrients, organic matter) may subsequently lead to greater sediment liberation and availability of these elements under reducing conditions (e.g., for Fe and Mn (ref.[1] and references therein), or SO_4^{2-} (ref. [15]). These effects can enhance AOM in surface sediments, which ultimately plays an important role in lowering ecosystem emissions rates[23,24].

Elevated ion content may restrict the delivery of fresh particulate organic matter needed to fuel sediment methanogenesis[20]. Under elevated ionic content and alkaline conditions, surface water productivity can also be restricted due to impacts on nutrient and micronutrient availability and possibly other factors[32–35]. The biomass of phytoplankton can be dramatically lower than expected based on total nutrient content, even at sub-saline conditions[32,33]. The mechanism(s) restricting primary production are complex, in some cases related to Fe complexation with organic matter that restricts bioavailable Fe for phytoplankton growth[34]. Intense deficiencies in surface water PO_4^{3-} (ref.[32]) and dissolved inorganic N [34] have been observed despite elevated TP and TN content. Limitations to N-fixation and bioavailable N production (ref.[34] and references therein) as well as complexation of P with Ca^{2+} [17], DOM and metals[35] may lead to bio-available macronutrient deficiencies and limited primary production. As salinity increases, constraints on primary production can have negative effects on fresh particulate organic matter deposition and organic substrate provision to sediment methanogens (which are not necessarily reflected in bulk DOC measurements that generally scale positively with CH_4 content in our regressions (Fig. 1, Table 1)). As such, salinity is a useful empirical predictor of CH_4 content in part because it reflects the geochemical limitations that can be imposed on primary producer growth and organic matter supply to methanogens.

Supporting Text S2. Analysis exploring the importance of within-site, cross-season, and cross-site replication for improving estimates of pCH_4.

Overview:

The objective of this analysis was to evaluate the relative importance of sources of uncertainty in our estimates of CH_4 content in small lentic systems in the Canadian Prairies, thereby informing the validity of our approach in the regional upscaling calculations. The upscaling calculations are conservative, first order approximations of the error that could be induced from using previous empirical relationships to estimate CH_4 emissions, versus the models generated in this paper using salinity as a key predictor. Our expectation was that between-site variability was more important to constrain than within-site variability in the context of regional upscaling, an expectation that was confirmed in this analysis.

Methods:

To assess the relative importance of within-site, cross-season, and cross-site variation to precision of site-level pCH_4 estimates, we used the peripheral wetland dataset where surface water pCH_4 was quantified alongside a minimal subset of other environmental parameters in 2021 in 47 sites across Alberta (AB), Saskatchewan (SK), and Manitoba (MB; 15-16 sites per province). Wetland ponds were sampled between two and 11 times (most sites sampled five times) over the open-water season and a subset of 18 ponds were sampled at four locations within the open-water area on each sampling occasion for a total of 472 measurements of pCH_4 over 198 unique site-date combinations.

We used a lognormal generalized linear mixed model (residual analyses supported this choice) with a fixed effect of province (AB, SK, or MB) and province-specific random effects of site(province), station(site), and date(station) to examine the relative importance of spatial and temporal sources of variation. Using model outputs, we then ran simulations to quantify how reallocating sampling effort would influence the precision of estimates of pCH_4. Because station-to-station variance was estimated to be zero for each province, we only compared effects of reallocation of sampling effort to number of sites vs frequency of site visits. For all simulation runs, total sampling effort was constrained to be close to total 2021 efforts (n = 468 to 471). Simulated pCH_4 values were generated from province-specific fixed estimates (i.e., βX) plus site-level and within-site error realizations by province (drawn from normal distributions with mean = 0 and variance = Site(Province) and within-site residual estimates, respectively). We used n = 30 simulation runs per sampling allocation scenario as results were consistent with larger-scale trials. We then re-ran lognormal generalized linear mixed models for all simulation iterations to obtain estimated marginal means and associated standard errors for the effect of province. We then calculated average (i.e., mean) estimated marginal means and standard errors across all 30 simulation runs. We compared the standard error of estimated marginal means at the province level across two sampling scenarios, with reductions in standard error indicating value for improving the precision of pCH_4 estimates at broad (i.e., provincial) scales.

Findings:

As summarized in Table S2, the station-to-station variance (i.e., variance in pCH_4 between samples from multiple locations within a site on a single sampling occasion) was estimated to be zero. Thus, sampling from multiple locations within a given wetland likely would not have

improved pCH_4 estimates in the primary dataset used to predict regional-scale patterns of CH_4 cycling. Except in Alberta, variance associated with sites was greater than variance associated with dates. Because the station variance component was zero, we focussed simulations on trade-offs between number of sites sampled and frequency of sampling. Given a constant total sampling effort, we estimated that there would be a 21-25% reduction in standard error achieved by doubling the number of basins sampled per province from 16 to 32 (at the expense of fewer measurements per basin; Tables S2,S3). We would expect a 30% reduction if all variation was attributed to basin-to-basin differences. Thus, the greatest improvements in precision and reduction in uncertainty of pCH_4 estimates at broad-scales are made through sampling more wetland systems less intensively (as in our primary dataset) compared with fewer wetlands sampled more frequently.

Ultimately, this exercise identifies that by sampling multiple small lentic systems (wetlands and ponds) across long environmental gradients (salt content, trophic status, etc.) as we have done and presented in figures 2 and 3, we have constrained more of the regional variance in surface water CH_4 content and thus emissions, than had we invested more resources in sampling multiple locations per system, at the cost of broader spatial coverage and inter-site sampling. Thus, while previous studies recommend highly-intensive sampling of a single ecosystem to fully constrain annual emissions patterns in a single ecosystem, it is clear that a lower-resolution approach that prioritizes multiple sites and repeated temporal sampling is a better approach where the aim is to constrain regional variability in emissions budgets.

While the goal of our paper was not to provide a completely refined emissions budget for Prairie Canada, this analysis does additionally provide an important road map for future research. The findings from this simulation exercise will help to guide study design where the aim is to

minimize the uncertainty in the overall, annual-scale emissions budget for lentic ecosystem CH_4 emissions at the regional scale.

Supporting References:

1. Deocampo, D. M. & Jones, B. F. Geochemistry of Saline Lakes. in *Treatise on Geochemistry* 437–469 (Elsevier, 2014). doi:10.1016/B978-0-08-095975-7.00515-5.
2. Last, W. M. & Ginn, F. M. Saline systems of the Great Plains of western Canada: an overview of the limnogeology and paleolimnology. *Saline Syst* **1**, 10 (2005).
3. Dillon, P. J. & Rigler, F. H. The phosphorus-chlorophyll relationship in lakes. *Limnol Oceanogr* **19**, 767–773 (1974).
4. Jeppesen, E., Peder Jensen, J., SØndergaard, M., Lauridsen, T. & Landkildehus, F. Trophic structure, species richness and biodiversity in Danish lakes: changes along a phosphorus gradient. *Freshw Biol* **45**, 201–218 (2000).
5. Filstrup, C. T. & Downing, J. A. Relationship of chlorophyll to phosphorus and nitrogen in nutrient-rich lakes. *Inland Waters* **7**, 385–400 (2017).
6. Guildford, S. J. & Hecky, R. E. Total nitrogen, total phosphorus, and nutrient limitation in lakes and oceans: Is there a common relationship? *Limnol Oceanogr* **45**, 1213–1223 (2000).
7. Bogard, M. J., St-Gelais, N. F., Vachon, D. & del Giorgio, P. A. Patterns of Spring/Summer Open-Water Metabolism Across Boreal Lakes. *Ecosystems* **23**, 1581–1597 (2020).
8. Karlsson, J. *et al.* Light limitation of nutrient-poor lake ecosystems. *Nature* **460**, 506–9 (2009).
9. del Giorgio, P. A. & Peters, R. H. Patterns in planktonic P:R ratios in lakes: Influence of lake trophy and dissolved organic carbon. *Limnol Oceanogr* **39**, 772–787 (1994).
10. Elser, J. J. *et al.* Shifts in Lake N:P Stoichiometry and Nutrient Limitation Driven by Atmospheric Nitrogen Deposition. *Science (1979)* **326**, 835–837 (2009).
11. Smith, V. H. Low nitrogen to phosphorus ratios favor dominance by blue-green algae in lake phytoplankton. *Science (1979)* **221**, 669–671 (1983).
12. Filstrup, C. T. *et al.* Evidence for regional nitrogen stress on chlorophyll *a* in lakes across large landscape and climate gradients. *Limnol Oceanogr* **63**, (2018).
13. Lapierre, J. F. & del Giorgio, P. A. Geographical and environmental drivers of regional differences in the lake pCO2 versus DOC relationship across northern landscapes. *J Geophys Res Biogeosci* **117**, 1–10 (2012).
14. Witherow, R. A. & Lyons, W. B. The fate of minor alkali elements in the chemical evolution of salt lakes. *Saline Syst* **7**, 2 (2011).
15. Margalef-Marti, R. *et al.* Upside down sulphate dynamics in a saline inland lake. *Sci Rep* **13**, 3032 (2023).
16. Camacho, A. *et al.* Methane Emissions in Spanish Saline Lakes: Current Rates, Temperature and Salinity Responses, and Evolution under Different Climate Change Scenarios. *Water (Basel)* **9**, 659 (2017).

17. Baldwin, D. S., Rees, G. N., Mitchell, A. M., Watson, G. & Williams, J. The short-term effects of salinization on anaerobic nutrient cycling and microbial community structure in sediment from a freshwater wetland. *WETLANDS* **26**, 455–464 (2006).

18. Wetzel, R. G. *Limnology: lake and river ecosystems*. (Academic Press, 2001).

19. Finlay, K. & Bogard, M. J. pH of Inland Waters. in *Encyclopedia of Inland Waters* 112–122 (Elsevier, 2022). doi:10.1016/B978-0-12-819166-8.00045-1.

20. Capone, D. G. & Kiene, R. P. Comparison of microbial dynamics in marine and freshwater sediments: Contrasts in anaerobic carbon catabolism1. *Limnol Oceanogr* **33**, 725–749 (1988).

21. Segarra, K. E. A., Comerford, C., Slaughter, J. & Joye, S. B. Impact of electron acceptor availability on the anaerobic oxidation of methane in coastal freshwater and brackish wetland sediments. *Geochim Cosmochim Acta* **115**, 15–30 (2013).

22. Bowles, M. W. *et al.* Remarkable Capacity for Anaerobic Oxidation of Methane at High Methane Concentration. *Geophys Res Lett* **46**, 12192–12201 (2019).

23. Martinez-Cruz, K. *et al.* Ubiquitous and significant anaerobic oxidation of methane in freshwater lake sediments. *Water Res* **144**, 332–340 (2018).

24. Segarra, K. E. A. *et al.* High rates of anaerobic methane oxidation in freshwater wetlands reduce potential atmospheric methane emissions. *Nat Commun* **6**, 7477 (2015).

25. Leu, A. O. *et al.* Anaerobic methane oxidation coupled to manganese reduction by members of the Methanoperedenaceae. *ISME J* **14**, 1030–1041 (2020).

26. Lu, Y.-Z. *et al.* Cr(VI) reduction coupled with anaerobic oxidation of methane in a laboratory reactor. *Water Res* **102**, 445–452 (2016).

27. Raghoebarsing, A. A. *et al.* A microbial consortium couples anaerobic methane oxidation to denitrification. *Nature* **440**, 918–921 (2006).

28. Herbert, E. R. *et al.* A global perspective on wetland salinization: Ecological consequences of a growing threat to freshwater wetlands. *Ecosphere* vol. 6 206 Preprint at https://doi.org/10.1890/ES14-00534.1 (2015).

29. Valenzuela, E. I. *et al.* Electron shuttling mediated by humic substances fuels anaerobic methane oxidation and carbon burial in wetland sediments. *Science of The Total Environment* **650**, 2674–2684 (2019).

30. Bogard, M. J., Donald, D. B., Finlay, K. & Leavitt, P. R. Distribution and regulation of urea in lakes of central North America. *Freshw Biol* **57**, 1277–1292 (2012).

31. Pham, S. V., Leavitt, P. R., McGowan, S. & Peres-Neto, P. Spatial variability of climate and land-use effects on lakes of the northern Great Plains. *Limnol Oceanogr* **53**, 728–742 (2008).

32. Sereda, J., Bogard, M., Hudson, J., Helps, D. & Dessouki, T. Climate warming and the onset of salinization: Rapid changes in the limnology of two northern plains lakes. *Limnologica* **41**, 1–9 (2011).

33. Robarts, R. D., Evans, M. S. & Arts, M. T. Light, Nutrients, and Water Temperature as Determinants of Phytoplankton Production in Two Saline, Prairie Lakes with High Sulphate Concentrations. *Canadian Journal of Fisheries and Aquatic Sciences* **49**, 2281–2290 (1992).

34. Evans, J. C. & Prepas, E. E. Relative importance of iron and molybdenum in restricting phytoplankton biomass in high phosphorus saline lakes. *Limnol Oceanogr* **42**, 461–472 (1997).

35. Waiser, M. J. & Robarts, R. D. Microbial nutrient limitation in prairie saline lakes with high sulfate concentration. *Limnol Oceanogr* **40**, 566–574 (1995).
